# Chest Radiography Optimization: Identifying the Optimal kV for Image Quality in a Phantom Study

**DOI:** 10.3390/jimaging12010049

**Published:** 2026-01-21

**Authors:** Ioannis Antonakos, Kyriakos Kokkinogoulis, Maria Giannopoulou, Efstathios P. Efstathopoulos

**Affiliations:** Department of Applied Medical Physics, Medical School, National and Kapodistrian University of Athens, 124 62 Athens, Greece; kyriakok@med.uoa.gr (K.K.); maragian@med.uoa.gr (M.G.); stathise@med.uoa.gr (E.P.E.)

**Keywords:** chest radiography, dose optimization, image quality, signal-to-noise ratio, contrast-to-noise ratio, entrance surface dose

## Abstract

Chest radiography remains one of the most frequently performed imaging examinations, highlighting the need for optimization of acquisition parameters to balance image quality and radiation dose. This study presents a phantom-based quantitative evaluation of chest radiography acquisition settings using a digital radiography system (AGFA DR 600). Measurements were performed at three tube voltage levels across simulated patient-equivalent thicknesses generated using PMMA slabs, with a Leeds TOR 15FG image quality phantom positioned centrally in the imaging setup. Image quality was quantitatively assessed using signal-to-noise ratio (SNR) and contrast-to-noise ratio (CNR), which were calculated from mean pixel values obtained from repeated acquisitions. Radiation exposure was evaluated through estimation of entrance surface dose (ESD). The analysis demonstrated that dose-normalized performance metrics favored intermediate tube voltages for slim and average patient-equivalent thicknesses, while higher voltages were required to maintain image quality in obese-equivalent conditions. Overall, image quality and dose were found to be strongly dependent on the combined selection of tube voltage and phantom thickness. These findings indicate that modest adjustments to tube voltage selection may improve the balance between image quality and radiation dose in chest radiography. Nevertheless, as the present work is based on phantom measurements, further validation using clinical images and observer-based studies is required before any modification of routine radiographic practice.

## 1. Introduction

Chest radiography is the most commonly performed radiographic examination worldwide and constitutes a substantial proportion of medical imaging procedures due to its broad diagnostic applicability and routine use in clinical practice [1,2,3]. Epidemiological evidence from both international and national studies consistently confirms its extensive utilization across diverse healthcare environments, including primary care services and screening programs [4,5].

Diagnostic information in chest radiography is derived from differences in X-ray attenuation among anatomical structures, making image quality critically dependent on sufficient contrast, spatial resolution, and acceptable noise levels. These factors directly affect the radiologist’s capacity to discriminate between subtle pathological findings and normal anatomical features [4]. Nevertheless, image quality is intrinsically associated with patient radiation exposure. Although the effective dose associated with a single chest radiograph is relatively low compared with other imaging modalities [6,7], the high examination frequency and the need for repeated imaging in patients with chronic conditions contribute to a non-negligible cumulative radiation burden at the population level [8,9,10,11,12]. Consequently, contemporary radiation protection frameworks, particularly the ALARA principle, underscore the necessity for continuous optimisation of radiographic protocols to achieve diagnostic adequacy while limiting unnecessary radiation exposure [1,13].

The entrance surface dose (ESD) represents a key dosimetric quantity for assessing patient exposure in radiographic procedures. It may be directly measured using appropriate dosimetric devices or indirectly estimated from X-ray tube output characteristics and exposure parameters [4,14]. Conventional optimisation strategies are largely based on manufacturer recommendations, professional expertise, and established diagnostic reference levels. However, growing evidence suggests that patient-related factors, especially chest thickness and body habitus, play a significant role in determining the optimal compromise between image quality and radiation dose [6,15,16,17,18,19]. Previous studies have shown that lower tube voltages may enhance lung detail visibility in smaller patients, whereas increased tube voltages are often required to ensure adequate penetration in patients with larger body size [17,18,19].

Despite the extensive body of literature addressing chest radiography optimisation, many published approaches depend on specialised equipment or anthropomorphic phantoms, which can limit their applicability in everyday clinical environments [20,21,22]. This highlights the need for simplified, clinically feasible optimisation methods that rely on readily available resources while still supporting informed parameter selection in routine practice.

The objective of the present study is to propose a simple and reproducible framework for optimising chest radiography acquisition parameters using basic phantom materials and standard image quality indicators. By systematically evaluating the relationship between tube voltage, patient-equivalent thickness, image quality metrics (SNR and CNR), and entrance surface dose, we introduce a practical strategy for adapting exposure settings to patient body habitus. Compared with our previous work, this study emphasizes a dose-normalized quantitative approach across multiple thickness categories using a unified quality index, enabling a systematic comparison that has not been previously reported.

## 2. Material and Methods

All exposures were performed using a ceiling-mounted digital radiography (DR) unit (AGFA DR 600; Agfa-Gevaert, Mortsel, Belgium) [23] equipped with a cesium iodide flat-panel detector. The system undergoes regular quality control according to national requirements and manufacturer recommendations. The detector has a nominal pixel size of 70 μm. All measurements were conducted in the Radiology Department of Attikon University Hospital (Athens, Greece).

The selected PMMA thickness ranges were chosen to approximate clinically realistic thoracic attenuation conditions corresponding to different adult body habitus categories (Table 1.), based on published correlations between chest thickness, body mass, and radiographic attenuation in adult populations [16].

**Figure 1:**
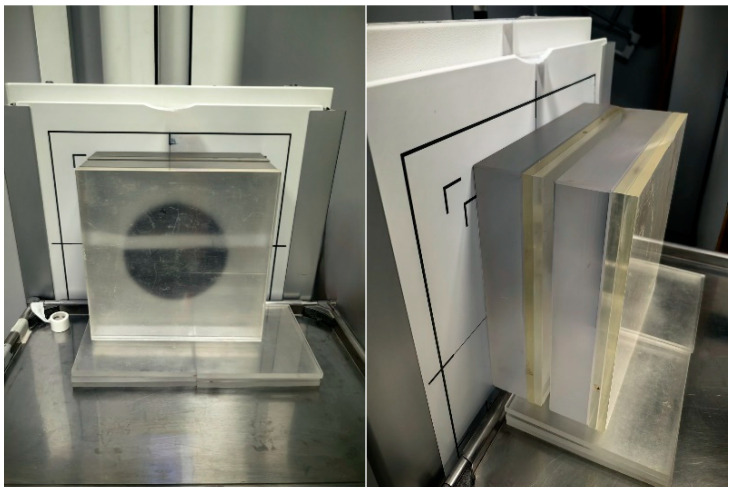
Measurement setup.

A Leeds TOR 18FG image quality phantom was placed centrally on the PMMA stack as demonstrated in Figure 1. The phantom includes low-contrast and high-contrast test objects, allowing quantitative assessment of SNR and CNR as well as qualitative evaluation of spatial resolution based on the number of resolvable detail patterns. The source-to-image distance (SID) was fixed at 180 cm, reflecting standard clinical practice. All exposures were taken in the posteroanterior configuration with the detector in the vertical Bucky stand (grid ratio 10:1, grid frequency 40 lines/cm). With automatic exposure control (AEC) disabled, tube current–time product (mAs) values were manually selected based on the institutional clinical protocol corresponding to each patient-equivalent thickness. The mAs values were subsequently adjusted as required by the system to maintain exposure index (EI) values within the manufacturer-recommended range.

For each patient-thickness category, the baseline tube voltage (kV) was selected according to the clinical protocol of the institution. Additional exposures were acquired by modifying the baseline kV by ±5 kV. For every exposure, the tube current-time product (mAs), exposure index (EI), and all acquisition parameters were recorded directly from the system console. Three repeated exposures were obtained for each configuration to ensure measurement stability.

### 2.1. Quantitative Image Quality Assessment

For a quantitative evaluation of image quality SNR and CNR were selected as primary metrics because they are widely used, system-independent indicators of image quality in projection radiography and are directly related to noise performance and low-contrast detectability, which are critical for chest imaging.

**Figure 2:**
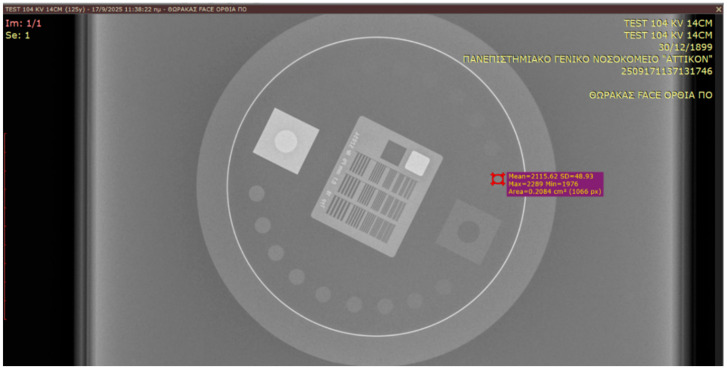
Leeds TOR 18FG image selection, MPV and SD evaluation.

SNR and CNR were calculated using circular regions of interest (ROIs) as illustrated in Figure 2:

- Signal ROI placed on the 10th low-contrast disk;
- Background ROI placed on a uniform region adjacent to the phantom.

For a quantitative evaluation of image quality, a calculation of contrast-to-noise ratio (CNR) and signal-to-noise ratio (SNR) was conducted. These metrics were derived as follows [24]:

$$CNR=\frac{\left|MPV-{MPV}_{bg}\right|}{\sqrt{\frac{{SD}^{2}}{2}+\frac{{SD}_{bg}^{2}}{2}}}$$

$$SNR=\frac{MPV}{SD}$$

see [25,26], where

- MPV is the mean pixel value from a circular ROI derived from the area of the tenth circular disk from each acquired image.
- MPV_bg_ is the mean pixel value from a circular ROI derived from the background area.
- SD and SD_bg_ are the standard deviations of the measurements.

In addition, the number of resolvable detail patterns of the Leeds test object was recorded as a qualitative indicator of spatial resolution.

### 2.2. ESD Calculation

Entrance Surface Dose (ESD) was calculated using Air Kerma measurement. (The measurement was conducted using), OCEAN software V. 4.5.0.0 (RTI Electronics AB, Sweden) together with Piranha X-ray meter (RTI Electronics AB, Sweden) [24] with serial No. BC1-05120015 that measures voltage, dose, dose rate, time and half-wavelength, calibrated on 14 March 2017, by Greek Atomic Energy Commission (EEAE).

The meter was placed on the radiography table at 100 cm SID (Source—Image Receptor Distance), using 15 × 15 cm^2^ field. Exposure parameters used were 115 kV and 2 mAs.

For the ESD calculation the following formula was implemented:

$$ESD=K_{air}\cdot \;{\left(\frac{d_{ref}}{d_{FSD}}\right)}^{2}\cdot \;a_{bsc}\cdot \;\frac{{mAs}_{ref}}{{mAs}_{m}}$$

see [14], where

- K_air_: air Kerma,
- d_ref_: reference distance of the measurement (100 cm),
- d_FSD_: Focus Surface Distance,
- a_bsc_: back scattering coefficient (1.35),
- mAs_ref_: the tube current used in this measurement (2 mAs),
- mAs_m_: the tube current used for each study measurement.

Potential sources of uncertainty include assumptions regarding the backscatter factor, positioning reproducibility, and the calibration status of the dosimetric system; however, these factors are not expected to significantly affect relative dose comparisons within the same experimental setup.

It should be emphasized that the reported ESD values represent relative entrance surface dose estimates under phantom-based conditions and are used exclusively for comparative optimization purposes, rather than as absolute patient skin dose measurements.

## 3. Results

Quantitative image quality metrics and radiation dose estimates were evaluated across all acquisition settings for the three simulated patient-equivalent thickness categories. Mean values derived from repeated measurements were used to examine trends in signal-to-noise ratio (SNR), contrast-to-noise ratio (CNR), and entrance surface dose (ESD) as a function of tube voltage selection.

For the slim-equivalent thickness configuration, SNR and CNR values exhibited a clear dependence on tube voltage, with intermediate voltage settings providing a favorable balance between image quality and radiation exposure (Table 2). When normalized to ESD, the combined performance indicators demonstrated higher values at lower tube voltages compared to higher settings, indicating more efficient image quality per unit dose under these conditions.

In the average patient-equivalent thickness configuration, both SNR and CNR showed moderate variation across the examined tube voltage range (Table 3). Dose-normalized performance metrics revealed optimal values at intermediate tube voltages, suggesting that these settings provided an effective compromise between noise suppression and radiation exposure.

For the obese-equivalent thickness configuration, image quality metrics were more strongly influenced by tube voltage selection (Table 4). Higher tube voltages were required to maintain adequate SNR and CNR values, reflecting increased attenuation associated with greater phantom thickness. Correspondingly, dose-normalized performance indices favored higher voltage settings in this category.

Across all thickness configurations, ESD values demonstrated systematic variation with acquisition parameters, reflecting protocol-driven exposure adjustments based on predefined mAs selections. Overall, the results highlight a consistent relationship between tube voltage selection, patient-equivalent thickness, and quantitative image quality metrics, underscoring the importance of thickness-specific optimization strategies in chest radiography.

The observed variation in ESD with increasing PMMA thickness is primarily attributable to increased X-ray attenuation and the corresponding exposure adjustments required to maintain detector signal levels under predefined protocol conditions.

**Figure 3:**
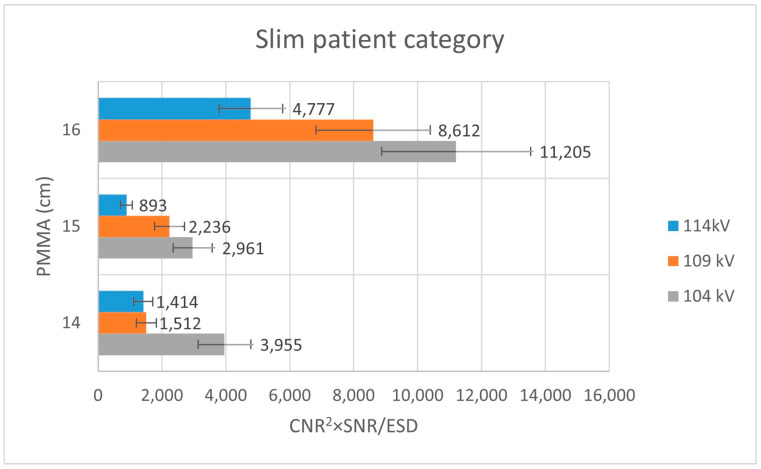
Bar plot illustrating the variation in the quality index (CNR^2^ × SNR/ESD) for the slim patient-equivalent group across different PMMA thicknesses, evaluated at three tube voltage settings (104 kV, 109 kV, and 114 kV).

**Figure 4:**
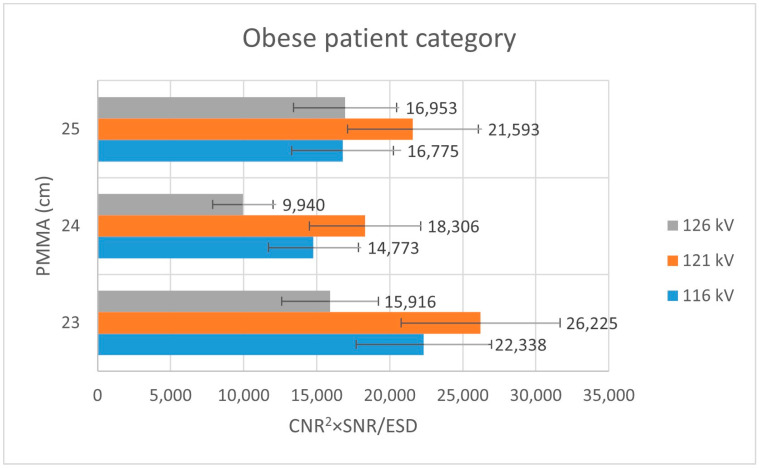
Bar plot depicting the quality index (CNR^2^ × SNR/ESD) for the obese patient-equivalent group across varying PMMA thicknesses, assessed at three tube voltage levels (116 kV, 121 kV, and 126 kV).

**Figure 5:**
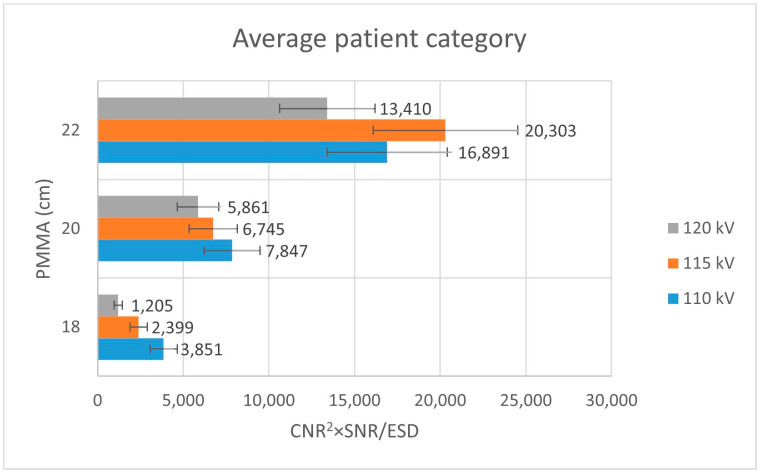
Bar plot illustrating the quality index (CNR^2^ × SNR/ESD) for the average patient-equivalent group as a function of PMMA thickness, evaluated at three tube voltage settings (110 kV, 115 kV, and 120 kV).

Analysis of Figure 3 shows that, within the thin patient-equivalent group, the quality index reaches its maximum at 104 kV—approximately 5 kV higher than the tube voltage currently applied in routine clinical practice—across all examined PMMA thicknesses. For the average patient-equivalent group (Figure 4), the highest quality index is observed at the standard clinical tube voltage for the greatest PMMA thickness (22 cm), whereas lower tube voltages yield superior performance for thinner phantom configurations. In contrast, for the obese patient-equivalent group (Figure 5), the quality index consistently attains its highest values at the standard tube voltage for all three PMMA thicknesses evaluated.

## 4. Discussion

In the present investigation, the results demonstrate that the optimal tube voltage varies markedly among patient-equivalent thickness groups, underscoring the importance of individualized acquisition strategies rather than reliance on uniform clinical protocols.

For slim patient-equivalent conditions, lower tube voltages (approximately 104 kV) achieved sufficient image quality while maintaining low entrance surface dose levels. This observation is consistent with reports indicating that lower tube voltage techniques can enhance image quality, particularly in lung imaging, and suggests that standard institutional protocols may lead to unnecessarily elevated radiation exposure in this patient group [15]. In average patient-equivalent conditions, thinner phantoms yielded improved performance at lower tube voltages, whereas increased thickness necessitated voltages closer to those routinely applied in clinical practice. In obese patient-equivalent scenarios, higher tube voltages systematically improved SNR and CNR values, albeit accompanied by a corresponding increase in ESD.

These findings are in agreement with previous studies showing that the relationship between tube voltage and diagnostic image quality is strongly modulated by patient size and anatomical region. Uffmann et al. [17], using flat-panel chest radiography, demonstrated that lower tube voltages (around 90 kVp) enhanced anatomical visibility compared with higher tube voltages when radiation dose was kept constant, particularly within the lung fields. Similarly, Grewal et al. [6] reported that carefully optimized reductions in exposure parameters can preserve diagnostic image quality. Additional phantom investigations have indicated that although increasing tube voltage may reduce entrance dose, it can also lead to contrast degradation unless compensatory beam filtration is employed [18]. The influence of patient thickness has been further supported by Monte Carlo simulations, which show that larger body sizes (20–28 cm) require higher tube voltages to maintain adequate image quality, and that digital radiography systems outperform computed radiography under equivalent exposure conditions [18]. Region-specific optimization has also been described, with lung fields exhibiting improved CNR at lower tube voltages, whereas mediastinal and spinal structures benefit from higher tube voltage settings [19]. The present results reflect the same overall pattern, with lean patient-equivalent conditions achieving adequate diagnostic quality at lower tube voltages, while obese-equivalent conditions required increased tube voltage to maintain comparable image adequacy.

While most published optimization studies advocate the use of anthropomorphic chest phantoms for digital radiography optimization [20,21,22], the present study demonstrates that meaningful preliminary optimization can be achieved using simple phantom materials commonly available in clinical environments. This approach enables an initial refinement of acquisition parameters aimed at improving image quality while limiting unnecessary radiation exposure.

The proposed composite quality index is not intended to replace observer-based performance studies or task-specific image quality evaluation. Instead, it is designed to serve as a practical, system-specific heuristic for comparative assessment of acquisition parameter combinations.

Several limitations of this study should be acknowledged. Although phantom-based measurements are widely employed, they cannot fully replicate patient anatomy or tissue heterogeneity. In addition, tube voltage was the only parameter systematically varied, while other influential factors, such as beam filtration, grid usage, and exposure time modulation, were not examined. Consequently, conclusions regarding absolute Exposure Index accuracy or inter-system EI comparability cannot be drawn from the present data. Furthermore, the investigation was conducted using a single digital radiography system, which may limit the generalizability of the findings to other manufacturers or detector technologies. Future work should extend this methodology to clinical populations and incorporate observer performance studies or diagnostic accuracy endpoints.

## 5. Conclusions

This phantom-based study demonstrated that tube voltage selection significantly influences the balance between image quality and radiation dose in digital chest radiography, with optimal settings strongly dependent on patient-equivalent thickness. The results indicate that lower or intermediate tube voltages may provide favorable dose-normalized image quality for slim and average patient-equivalent conditions, whereas higher tube voltages are required to maintain adequate image quality in obese-equivalent scenarios.

The findings highlight the importance of thickness-specific optimization strategies rather than uniform acquisition protocols. However, given the experimental nature of the study and the use of simplified phantom models, the results should be interpreted with caution. Future investigations incorporating clinical images, automatic exposure control behavior, and observer-based image quality assessment are required before translation of these optimization approaches into routine clinical practice.

## Figures and Tables

**Table 1:** PMMA thicknesses used and their corresponding patient categories.

PMMA Thickness (cm)	Patient Category
14	Slim (45–55 kg)
15	
16	
18	Average (65–85 kg)
20	
22	
23	Obese (90– 100kg)
24	
25	

**Table 2:** Data for Slim Patient category.

Slim Patient Category											
kV	mAs	d (cm)	EI	MPV	MPVbg	SD	SD_bg_	SNR	CNR	ESD ± SD (mGy)	CNR^2^ × SNR/ESD ± SD
104	3.7	14	215	2525.69	2421.13	57.75	49.49	43.73489	1.94426	0.042 ± 0.006	3955 ± 823
	4.2	15	208	2494.22	2406.24	60.43	47	41.27453	1.625253	0.037 ± 0.006	2961 ± 616
	4.8	16	193	2541.03	2394.76	56.91	45.31	44.64997	2.843615	0.032 ± 0.005	11,205 ± 2331
109	3.1	14	214	2521.57	2441.36	62.47	52.8	40.36449	1.386818	0.051 ± 0.008	1512 ± 314
	3.6	15	207	2469.56	2395.74	53.22	47.79	46.40286	1.45953	0.044 ± 0.007	2236 ± 465
	4	16	192	2556.29	2411.98	56.35	48.36	45.36451	2.748385	0.040 ± 0.006	8612 ± 1791
114	2.7	14	51	2496.26	2400.02	62.06	56.02	40.22333	1.627953	0.075 ± 0.011	1414 ± 294
	3.2	15	48	2466.51	2406.23	58.48	44.46	42.17698	1.160454	0.064 ± 0.010	893 ± 186
	3.5	16	189	2531.81	2394.26	58.93	48.79	42.96301	2.542603	0.058 ± 0.009	4777 ± 994

**Table 3:** Data for Average Patient category.

Average Patient Category											
kV	mAs	d (cm)	EI	MPV	MPVbg	SD	SD_bg_	SNR	CNR	ESD ± SD (mGy)	CNR^2^·SNR/ESD ± SD
110	5.1	18	170	2096.16	2170	47.68	42.8	43.96309	1.629815	0.030 ± 0.005	3851 ± 801
	6.5	20	148	2172.73	2273.55	49.95	47.34	43.4981	2.071821	0.024 ± 0.004	7847 ± 1632
	8	22	56	2269.12	2410.93	53.25	49.12	42.61258	2.768286	0.019 ± 0.003	16,891 ± 3513
115	4.4	18	167	2088.21	2156.89	50.35	44.42	41.47388	1.446575	0.036 ± 0.005	2399 ± 499
	5.6	20	56	2165.79	2259.97	46.87	45.6	46.20845	2.036793	0.028 ± 0.004	6745 ± 1403
	6.8	22	56	2255.51	2402.68	48.39	43.67	46.61108	3.193069	0.023 ± 0.004	20,303 ± 4223
120	3.7	18	160	2091.25	2157.52	54.03	47.02	38.70535	1.308483	0.055 ± 0.008	1205 ± 251
	4.7	20	53	2179.51	2288.37	47.23	45.6	46.14673	2.345001	0.043 ± 0.006	5861 ± 1219
	5.7	22	71	2245.88	2401.12	48.54	47.98	46.26864	3.216689	0.036 ± 0.005	13,410 ± 2789

**Table 4:** Data for Obese Patient category.

Obese Patient category											
kV	mAs	d (cm)	EI	MPV	MPVbg	SD	SD_bg_	SNR	CNR	ESD ± SD (mGy)	CNR^2^·SNR/ESD ± SD
116	7.3	23	55	2284.68	2438.84	48.39	48.99	47.21389	3.166093	0.021 ± 0.003	22,338 ± 4646
	8.4	24	113	2337.94	2451.56	49.27	45.57	47.45159	2.394214	0.018 ± 0.003	14,773 ± 3073
	9.2	25	103	2373.73	2496.38	49.79	51.06	47.67483	2.432132	0.017 ± 0.003	16,775 ± 3489
121	6.2	23	114	2246.03	2434.35	48.63	50.01	46.1861	3.817956	0.026 ± 0.004	26,225 ± 5455
	7.2	24	110	2330.56	2467.02	46.35	49.79	50.28177	2.836961	0.022 ± 0.003	18,306 ± 3808
	7.9	25	100	2361.27	2500.15	47.05	47.29	50.1864	2.944235	0.020 ± 0.003	21,593 ± 4491
126	5.3	23	77	2277.37	2445.64	47.6	46.56	47.84391	3.573911	0.038 ± 0.006	15,916 ± 3310
	6.1	24	107	2331.43	2443.31	45.39	42.63	51.3644	2.540901	0.033 ± 0.005	9940 ± 2068
	6.7	25	98	2367.91	2511.07	45.15	46.22	52.4454	3.133418	0.030 ± 0.005	16,953 ± 3528

## Data Availability

The original contributions presented in this study are included in the article. Further inquiries can be directed to the corresponding author.
